# Ten-year trends in the treatment and intervention timing for patients with metastatic spinal tumors: a retrospective observational study

**DOI:** 10.1186/s13018-022-03496-5

**Published:** 2023-01-10

**Authors:** Ryosuke Hirota, Atsushi Teramoto, Noriyuki Iesato, Mitsumasa Chiba, Toshihiko Yamashita

**Affiliations:** Department of Orthopaedic Surgery, Sapporo Medical University School of Medicine, S1W17, Chuo-Ku, Sapporo, Hokkaido 060-8556 Japan

**Keywords:** Metastatic spinal tumors, Walking ability, Time from symptom

## Abstract

**Background:**

Cancer treatment has recently evolved due to the advances in comprehensive therapies, including chemotherapy and radiotherapy. The aspect of cancer-related bone metastasis has undergone a paradigm shift with the transformation of orthopedic interventions for spinal metastasis. We performed this retrospective observational study to investigate the changes in patient status and metastatic spine-tumor treatment over the past decade.

**Methods:**

We included 186 patients (122 men and 64 women; mean age: 67.6 years) who were referred to our hospital between 2009 and 2018 and were diagnosed and treated for metastatic spinal tumors. We classified the patients into early (81 patients from 2009 to 2013) and late (105 patients from 2014 to 2018) groups. The following components were investigated and compared between the groups: primary tumor, time taken from subjective-symptom onset to hospital visit, primary tumor evaluation during the visit, walking capacity due to lower paralysis during the visit, local treatment details, and post-treatment functional prognosis.

**Results:**

Predominant primary tumors with similar trends in both groups included lung cancer, multiple myeloma, and prostate cancer. The percentage of non-ambulatory patients during the consultation was significantly lower in the late group (28% vs. 16%, *P* = 0.04). Among non-ambulatory patients at the time of hospital visit, the mean time from the primary doctor consultation to our hospital visit was 2.8 and 2.1 days in the early and late groups, respectively. In both groups, surgical procedures were performed promptly on the non-ambulatory patients; however, postoperative lower function did not improve in approximately half of the patients.

**Conclusions:**

Our findings demonstrated that in recent years, patients tended to be referred promptly from their previous doctors under a favorable collaboration system. However, the effectiveness of lower paralysis treatment remains limited, and it is important to raise awareness regarding the importance of early consultation among the general public for earlier detection.

## Background

The treatment of metastatic spine tumors has undergone a major paradigm shift in recent years. First, multidisciplinary and systemic therapies, such as anticancer agents, molecular-targeted agents, bisphosphonate therapy, and radiotherapy, are currently available for the treatment of cancer, thus improving mortality. Aaron et al. reported that chemotherapy, according to genetic mutations, prolongs the overall survival and progression-free survival in patients with advanced cancer [1]. Birgitt et al. noted that the recently developed high-precision radiation therapy is expected to provide superior tumor control [2]. With these advances in comprehensive treatment, the patient’s life expectancy is increasing, and the number of patients who develop metastases is also increasing.

Spinal metastases account for 50% of all cancer-related bone metastases, and the number of patients with spinal metastases is also increasing [3]. Second, diversification and advancement of surgical therapies have led to the active use of patient-tailored surgeries. Minimally invasive spinal stabilization (MIST) using percutaneous pedicle screws [4, 5] and total en bloc spondylectomy (TES) [6, 7] for a local cure have been applied for treating metastatic spinal tumors.

In the treatment of metastatic spine tumors, it is important to consider the degree of priority and balance with the treatment of the primary organ and other organs, delay the onset of pathological fractures and paralysis to the maximum extent possible, and secure life by maintaining the quality of life (QOL) as much as possible. However, the timing and type of treatment initiation vary depending on the patient and institution; therefore, no consensus has been established.

Therefore, we investigated the changes in the treatment type and intervention timing for metastatic spinal tumors to elucidate the treatment trends and identify future issues.

## Methods

### Study design

A retrospective analysis of single-center observational data.

### Patient population

A total of 186 patients who were referred to our hospital, were diagnosed with metastatic spinal tumors, and received treatment between 2009.4.1 and 2019.3.31 were eligible in this study. Finally, 122 men and 64 women (mean age: 67.6 years) were included in the study.

The primary lesion, evaluation of the primary lesion at the time of consultation, referral hospital, time-lapse between the subjective-symptom onset and visit to our hospital, local treatment details, and pre- and post-treatment mobility were investigated. The evaluation of pre- and post-treatment mobility was performed in patients within 2 months of treatment. The functional outcome was the ability to walk independently at 2 months after treatment. The spinal cord independence measure (SCIM) indoor mobility item was used to assess independent walking, with a score of 4–8 implying the ability to walk independently indoors. The SCIM is a validated tool specifically designed to assess the patient’s functional capacity following SCI [8]. Previous studies have demonstrated that SCIM indoor mobility outcomes are strongly correlated with the SCIM’s moderate and outdoor distance mobility outcomes [9, 10]; therefore, we selected indoor mobility as a general representative of the total mobility outcomes. These items were compared between the groups treated in 2009–2013 (early) and those treated in 2014–2018 (late).

### Surgical procedure

In cases where the tumor was confined to the vertebral arch with no evidence of spinal instability, only posterior decompression was performed. In most cases, posterior decompression and fusion were performed using a conventional technique in which a vertebral root screw was inserted 2 above-2 below for fusion, and then a laminectomy was performed at the level of the affected vertebral body to decompress the neural tissue. If bleeding could be controlled, ventral dural lesions were resected posterolaterally as much as possible by resection of the vertebral arch root. TES was performed in patients with a single tumor and good long-term prognoses.

### Statistical analysis

Student’s *t*-test, chi-squared test, and Fisher’s exact test were used to compare the continuous, discontinuous, and categorical variables, respectively. Statistical significance was set at *P* < 0.05. All statistical analyses were conducted using SPSS (version 23.0; IBM Corporation, Armonk, NY, USA).

## Results

**Fig. 1:**
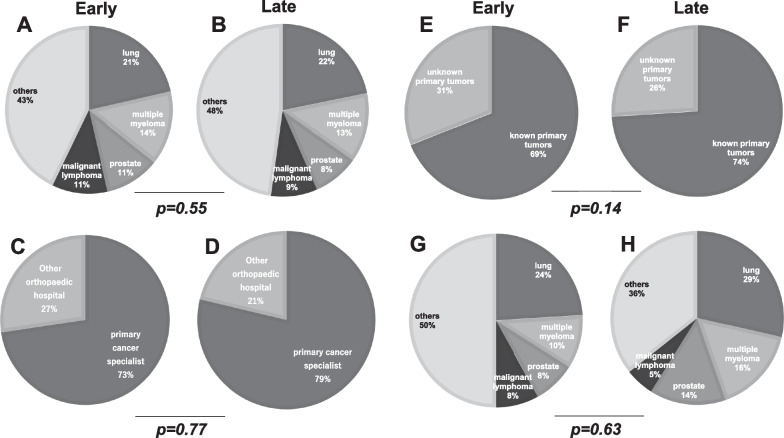
Comparison of the primary tumor, referral source, primary tumor evaluation at the time of consultation, and primary site in patients with unknown primary tumors between the early and late groups. **A** primary tumor of the early group, **B** primary tumor of the late group, **C** referral source of the early group, **D** referral source of the late group, **E** evaluation of the primary tumor of the early group, **F** evaluation of the primary tumor of the late group, **G** primary site in patients with unknown primary tumors of the early group, and **H** primary site in patients with unknown primary tumors of the late group

In total, 81 and 105 patients were treated in the early and late groups, respectively. Tables 1and 2 show the patient characteristics. The mean ages of the two groups were 66.5 (early group) and 68.4 (late group) years, respectively, with no significant difference between them (*P* = 0.45). At the primary site, lung cancer, multiple myeloma, prostate cancer, and breast cancer were the predominant conditions in both groups (Fig. 1A, B).

### Referral source

In the early and groups, 70–80% of patients were referred from a primary cancer specialist to our hospital (Fig. 1C, D).

### Evaluation of the primary tumor at the time of consultation

The percentage of patients with known primary tumors at the time of consultation was 69.2% in the early group and 73.8% in the late group; however, the difference between both groups was not statistically significant (P = 0.14) (Fig. 1E, F).

### Primary site in patients with unknown primary tumors

The proportion of patients with unknown primary tumors was high in those with lung cancer, multiple myeloma, prostate cancer, and malignant lymphoma as primary tumors in both groups (Fig. 1G, H).

### Treatment details

**Fig. 2:**
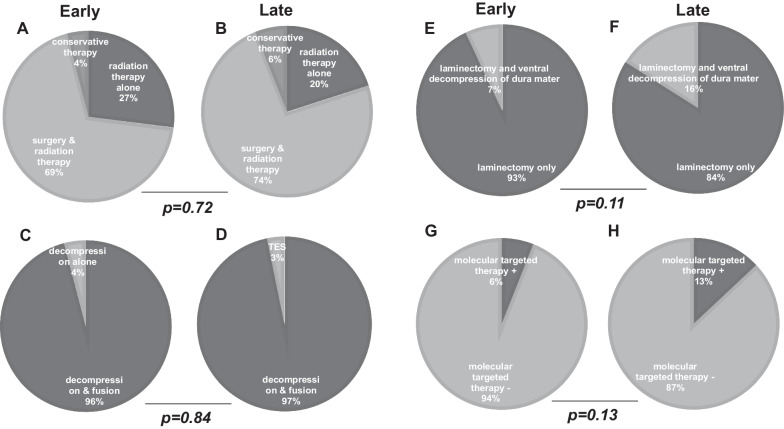
Comparison of treatment details. **A** early group treatment, **B** late group treatment, **C** surgical procedure of the early group, **D** surgical procedure of the late group, **E** spinal cord decompression of the early group, **F** spinal cord decompression of the late group, **G** availability of molecular-targeted therapy of the early group, **H** availability of molecular-targeted therapy of the late group

In the early and late groups, surgical treatment and radiotherapy were often combined, and radiotherapy was exclusively used in approximately 20% of patients (Fig. 2A, B). The majority of operated patients in both groups underwent decompression and fusion using the open method (Fig. 2C, D). A greater proportion of patients in the late group underwent anterior dural decompression in addition to laminectomy compared to the early group (7.40% vs. 16.2%), although with no significant difference between the groups (*P* = 0.11) (Fig. 2E, F).

### Pre and postoperative molecular-targeted therapy

The percentage of patients for whom molecular-targeted therapy was indicated and performed before or after surgery was 6.17% in the early group and 13.3% in the late group. The difference between the two groups was not statistically significant (*P* = 0.13) (Fig. 2G, H).

### Walking ability before and after treatment

Table 3 shows the patient characteristics of those who would walk after treatment. Of the 186 patients, 111 patients (59.7%) had an assessable walking function at 2 months after treatment; however, we could confirm their mobility after treatment.

**Table 1:** General characteristics of all patients

	*n* = 186
Age (years)	67.7 ± 10.6
Male (no. of patients)	122 (64.6%)
BMI (kg/m^2^)	20.1 ± 3.2
*Primary tumor (no. of patients)*	
Lung Multiple myeloma Prostate Malignant lymphoma Breast Liver others	39 (21.0%) 27 (14.5%) 21 (11.3%) 19 (10.2%) 10 (5.4%) 9 (4.8%) 61 (32.8%)
Spinal level *(no. of patients)*	
Cervical Thoracic Lumbar	16 (8.6%) 79 (42.5%) 91 (48.9%)
Time from symptom onset to visit at our hospital (day)	26.4 ± 10.5
Walkable before treatment (%)	146 (79.0%)

**Table 2:** Patient characteristics of the Early and Late groups

	Early group *n* = 81	Late group *n* = 105	*P*-value
Sex (male:female)	53:28	69:36	0.783^b^
Age (years)	66.5 ± 12.7	68.4 ± 11.8	0.577^a^
Metastasis level (n)	Cervical, 7 Thoracic, 34 Lumbosacral, 40	Cervical, 9 Thoracic, 45 Lumbosacral, 51	0.515^b^

Values are expressed as means ± standard deviation or percentages

^a^Student’s *t* test

^b^χ^2^ test

**Table 3:** Patient characteristics we could confirm regarding mobility after treatment

	Early group *n* = 50	Late group *n* = 61	*P*-value
Sex (male:female)	31:19	36:25	0439^b^
Age (years)	67.7 ± 15.3	69.1 ± 14.2	0.381^a^
Metastasis level (*n*)	Cervical, 6 Thoracic, 18 Lumbosacral, 26	Cervical, 4 Thoracic, 25 Lumbosacral, 31	0.299 ^b^

Values are expressed as mean ± standard deviation or percentages

^a^Student’s *t* test

^b^χ^2^ test

**Fig. 3:**
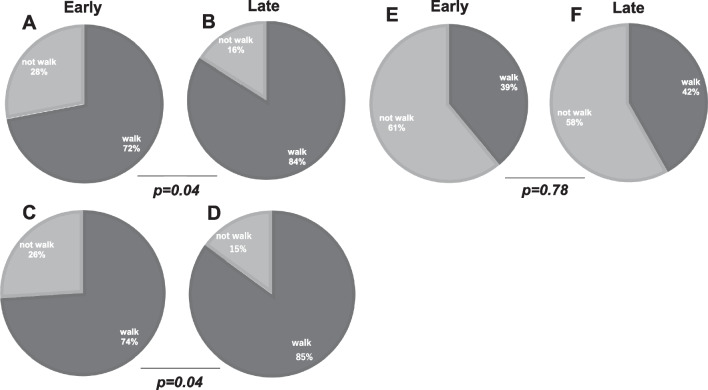
Comparison of mobility pre- and post-treatment. **A** mobility of the early group at the time of intervention, **B** mobility of the late group at the time of intervention, **C** mobility after treatment in all patients of the early group, **D** mobility after treatment in all patients of the late group, **E** mobility after treatment in patients who could not walk before treatment of the early group, and **F** mobility after treatment in people who could not walk before treatment of the late group

The follow-up rate was not significantly different between the early (61.7%) and late groups (58.1%). The percentage of patients who could walk before treatment was significantly higher in the late group (83.6%) than in the early group (73.2%) (*P* = 0.04) (Fig. 3A, B). The percentage of patients who were able to walk at 2 months after treatment was significantly higher in the late group (85.2%) than in the early group (74.0%) (*P* = 0.04) (Fig. 3C, D). Nevertheless, more than 50% of the patients who could not walk before treatment remained unable to walk after treatment (Fig. 3E, F), a finding that was similar in both the groups.

### Time from symptom onset to visit at our hospital

**Fig. 4:**
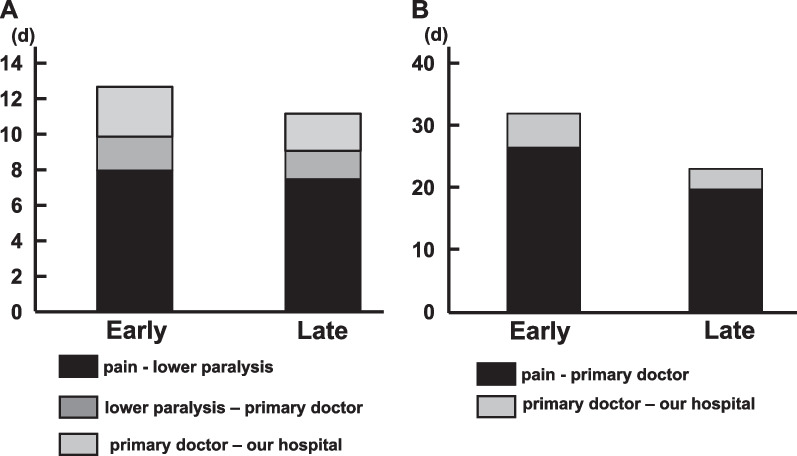
Time from the onset of symptoms to our hospital visit. **A** unwalkable patients at the time of visit to our hospital and **B** walkable patients at the time of visit to our hospital

Figure 4 shows the time from the symptom onset to our hospital visit. The average time from pain onset to lower paralysis onset in patients who were unable to walk at the time of hospital visit was approximately 8 days, which was the same for both groups. In contrast, the time taken from primary doctor consultation to our hospital visit was 2.8 and 2.1 days in the early and late groups, respectively, and patients in the late group tended to visit our hospital earlier (Fig. 3A). For patients who could walk at the time of their hospital visit, it took 26.5 and 19.8 days from the onset of pain to visit the primary doctor for patients in the early and late groups, respectively (Fig. 3B).

## Discussion

In this study, the lung, breast, renal, and prostate cancers were the predominant primary sites, as observed in previous reports, including cases where the primary site was unknown at the time of treatment initiation. This trend was similar in the early and late groups, and patients with a history of these cancers should be wary about spinal metastasis.

In this study, the time between the previous doctor’s visit and a visit to our hospital decreased in the preceding 5 years, and the percentage of non-ambulatory patients at the time of visit to our hospital decreased. This may be due to the increasing momentum of multidisciplinary treatment in recent years, and the requirement for early intervention for spinal metastases has been recognized by the primary doctors. Nonetheless, the effect of surgical treatment on patients who developed paralysis was limited in both groups, and more than 50% of patients were unable to regain ambulation. Patchell et al. [11] reported that in a randomized controlled trial of 101 patients with spinal metastases and neurological symptoms, the surgical results were significantly superior to those who underwent exclusive radiotherapy. Rades et al. [12] performed a matched-pair analysis of 108 patients and reported that the results of combined surgery and exclusive radiotherapy were comparable. The radiosensitivity of carcinoma varies, and the mode of spinal cord compression, patient’s general condition, and prognosis for life are also extremely variable; therefore, a decision regarding the treatment strategy often depends on the individual case. At our hospital, patients with spinal cord symptoms were treated with posterior decompression and fusion in addition to radiotherapy, provided their general condition allowed for these treatments. MIST, using percutaneous pedicle screws [4, 5] and percutaneous vertebroplasty [13, 14], was recently introduced. In a systematic review by Mendel et al., percutaneous vertebroplasty was found to be effective in treating metastatic spinal tumors [15].

These surgical interventions are based on the idea that, as a merit, they can indirectly prolong life by improving the activities of daily living (ADL) through surgery and increasing the probability of receiving postoperative adjuvant therapy. However, minimally invasive procedures are particularly beneficial to patients who are not paralyzed and do not require decompression. In cases where spinal cord compression is strong and paralysis appears, posterior decompression and resection of the tumor must be performed in combination, considering the possibility of postoperative paralysis flaring up or worsening due to tumor growth. Grade 3 on the epidural spinal cord compression scale [16] indicates the requirement for posterior decompression or tumor resection in principle [17]. In recent years, TES, a radical surgery for spinal metastases, has also been reported to improve the local control of metastases and prognosis of life in certain carcinomas, depending on the primary tumor stage [18, 19]. However, this treatment is not indicated when the tumor is advanced and has multiple metastases.

Radiation therapy should be initiated as soon as the diagnosis of metastatic spinal tumors is confirmed. The National Institute for Health and Clinical Excellence guidelines recommend that radiotherapy for spinal metastases should be initiated within 24 h after diagnosis [20].

On this premise, it is clear that surgical or radiation treatment should be performed as early as possible before the appearance of lower paralysis symptoms.

In this study, the referral time from the primary doctor consultation to our hospital was shortened; however, it took a relatively long time from the onset of pain to the primary doctor visit. In particular, in patients with pain as the only symptom, it took 26.5 and 19.8 days for the patients in early and late groups to consult the primary doctor, respectively. Even in cases where lower paralysis appeared, it did not occur suddenly; in many cases, pain occurred in the spinal metastases as a prodromal symptom a few days before the onset of paralysis. When paralysis occurs due to spinal metastasis, ADL and QOL decrease significantly. If the performance status is reduced due to paralysis, aggressive treatment, such as chemotherapy, cannot be performed, rendering it difficult to improve the prognosis. Even though there are several treatment options, early detection remains considerably crucial. In this study, molecular-targeted therapy was administered more frequently in the late group, which may have contributed to the recovery of postoperative gait function. Advanced medical treatments, such as molecular-targeted therapy and immunotherapy, have significantly developed in recent years, especially in lung and breast cancers [21, 22], and may expand the indications for surgery for patients with spinal metastases in future. Early detection of spinal metastasis and timely intervention are therefore necessary. In light of the above, raising awareness of spinal metastases in patients who experience pain that appears without a trigger and persists for several days and encouraging these patients to seek medical attention are potentially effective.

In this study, we investigated the changes in the patient status and treatment of metastatic spinal tumors over the past 10 years. This study has certain limitations. We did not include cases treated with advanced radiation therapy, such as heavy particle therapy, stereotactic body radiation therapy, and intraoperative radiation therapy. Although the usefulness of these therapies for metastatic spinal cord tumors has been reported in recent years [23, 24], reports of their benefit to patients with respect to gait function are scanty, and future studies are warranted. In addition, it was a retrospective study, the primary carcinoma was not analyzed in detail, and the long-term results of the patients were not followed. Few studies have investigated the evolution of treatment and patient status at the initiation of treatment for metastatic spine tumors, which are subjected to ever-changing treatment strategies. In recent years, patients with metastatic spine tumors have been promptly referred by their primary doctors under a favorable medical cooperation system. However, the effectiveness of the treatment of patients with lower paralysis remains limited, and it is desirable to create a system for earlier detection.

## Conclusion

In recent years, patients tended to be referred promptly from their previous doctors under a favorable collaboration system. However, the effectiveness of lower paralysis treatment remains limited, and it is important to raise awareness regarding the importance of early consultation among the general public for earlier detection.

## Data Availability

Not applicable.
